# The lung mesenchyme in development, regeneration, and fibrosis

**DOI:** 10.1172/JCI170498

**Published:** 2023-07-17

**Authors:** Elie El Agha, Victor J. Thannickal

**Affiliations:** 1 Department of Medicine V, Internal Medicine, Infectious Diseases and Infection Control, Universities of Giessen and Marburg Lung Center (UGMLC), German Center for Lung Research (DZL), Justus-Liebig University Giessen, Giessen, Germany.; 2 Cardio-Pulmonary Institute (CPI), Giessen, Germany.; 3 Institute for Lung Health (ILH), Giessen, Germany.; 4 John W. Deming Department of Medicine, Tulane University School of Medicine, New Orleans, Louisiana, USA.; 5 Southeast Louisiana Veterans Health Care System, New Orleans, Louisiana, USA.

## Abstract

Mesenchymal cells are uniquely located at the interface between the epithelial lining and the stroma, allowing them to act as a signaling hub among diverse cellular compartments of the lung. During embryonic and postnatal lung development, mesenchyme-derived signals instruct epithelial budding, branching morphogenesis, and subsequent structural and functional maturation. Later during adult life, the mesenchyme plays divergent roles wherein its balanced activation promotes epithelial repair after injury while its aberrant activation can lead to pathological remodeling and fibrosis that are associated with multiple chronic pulmonary diseases, including bronchopulmonary dysplasia, idiopathic pulmonary fibrosis, and chronic obstructive pulmonary disease. In this Review, we discuss the involvement of the lung mesenchyme in various morphogenic, neomorphogenic, and dysmorphogenic aspects of lung biology and health, with special emphasis on lung fibroblast subsets and smooth muscle cells, intercellular communication, and intrinsic mesenchymal mechanisms that drive such physiological and pathophysiological events throughout development, homeostasis, injury repair, regeneration, and aging.

## Introduction

Over the past century, there has been a remarkable shift in disease burden from acute/subacute communicable diseases to more chronic noncommunicable diseases. The reasons for this shift are likely multifactorial, related to improved prevention and treatment of acute infectious diseases, increasing socialization and industrialization, lifestyle changes that contribute to metabolic disorders, and the aging of populations worldwide. Even with the recent viral pandemic, a notable proportion of survivors develop chronic, noncommunicable clinical syndromes following recovery from the acute infectious disease. Many of these chronic diseases of the 21st century can be attributed to loss of homeostatic maintenance and regeneration or inefficient/incomplete repair of affected tissues and organs. This often manifests as tissue fibrosis in diverse organ systems such as the liver, kidney, heart, and lungs. A fundamental organizing principle in the structural engineering of these tissues/organs during development is the close apposition of the epithelium with mesenchymal cells or fibroblasts that function as signaling hubs and stem cell niches to not only maintain homeostasis, but support the repair/regeneration of these tissues following injury.

The mammalian lung can be divided into two main compartments based on cellular composition and the surrounding tissue microenvironment: the epithelium, which constitutes the internal lining, and the stroma, which comprises the connective tissue and vasculature. The connective tissue predominantly consists of mesenchymal cells and extracellular matrix (ECM). While the lineage hierarchy and functional characteristics of epithelial cells have been well investigated during lung development and maturation, and to a certain extent in aging and disease, a similar understanding of the stromal compartment is still lagging. This is mainly due to a gap in our knowledge regarding mesenchymal cell heterogeneity and hierarchy, particularly in terms of cellular identity and plasticity, and how this translates to adaptive versus maladaptive tissue repair in physiological and pathophysiological settings, respectively. In this Review, we will focus our discussion on mesenchymal cell functions in support of epithelial repair/regeneration, and refer readers interested in a more expanded understanding of endothelial cell regeneration and vascular repair, the role of angiocrine-derived factors in epithelial regeneration, and mesenchymal-endothelial interactions to other recently published reviews as well as original research articles (1–6).

The epithelium of the lung is populated by specialized cell types along the proximo-distal axis, such as basal, club, and ciliated cells in the conducting airways and alveolar epithelial type 1 (AT1) and type 2 (AT2) cells in the smallest respiratory units of the lungs, the alveoli. Although the adult lung generally possesses a low cell turnover rate, specialized epithelial populations are engaged during repair after injury, including subsets of differentiated cells with stem/progenitor cell capacity such as basal cells, club cells, and AT2s, and cells in transitional states such as p63^+^ KRT5^+^ pods, KRT8^+^ basaloid cells, pre-AT1 transitional cell state (PATS), and interleukin-1 receptor–positive (IL-1R^+^) damage-associated transient progenitors (DATPs) (7–23). Interestingly, the mesenchymal counterpart that constitutes the niche for such epithelial subsets is poorly characterized at the spatial, cellular, and molecular levels. This is a major limitation given that the lung mesenchyme plays an indispensable role in instructing epithelial cell behavior not only during lung development but also during repair after injury, and — presumably — in dysmorphogenic events that lead to aberrant repair and chronic, predominantly progressive and fatal lung diseases.

**Figure 1:**
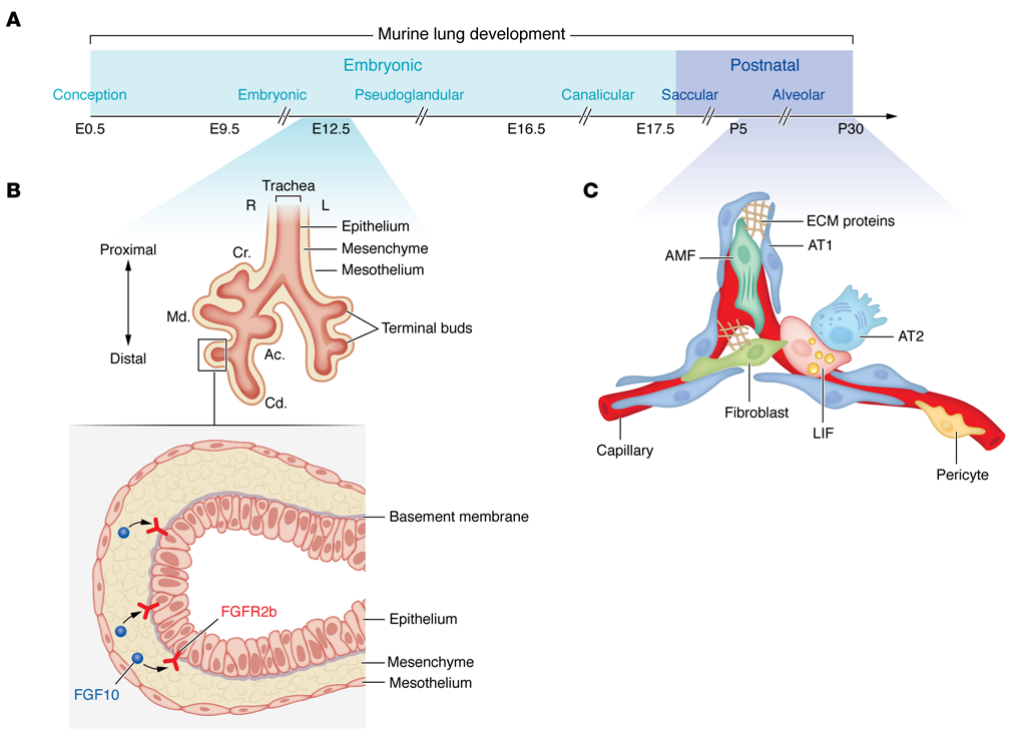
Overview of pre- and postnatal lung development. (**A**) Lung development consists of an embryonic and postnatal phase. The different stages of mouse lung development are shown through P30. (**B**) A schematic representation of an embryonic lung at E12.5 and a corresponding sagittal section of a distal epithelial bud with the surrounding mesenchymal tissue. FGF10/FGFR2b signaling is highlighted. (**C**) A schematic representation of an alveolus with the constituent cell types during the alveolar stage of lung development. Cr., cranial lobe; Md., medial lobe; Cd., caudal lobe; Ac., accessory lobe; AMF, alveolar myofibroblast; AT1, alveolar epithelial type 1; AT2, alveolar epithelial type 2; LIF, lipofibroblast.

During embryonic lung development, the interplay between the primitive endoderm and splanchnic mesoderm is pivotal for lung bud formation and subsequent branching morphogenesis, an event that marks the pseudoglandular stage of lung development (reviewed in refs. 24–27) (Figure 1, A and B). During later developmental stages, a similar mode of crosstalk is instrumental for the formation of mature differentiated epithelial cells such as AT1s and AT2s, as well as mesenchymal cells such as airway and vascular smooth muscle cells (ASMCs and VSMCs), alveolar myofibroblasts (AMFs), and lipofibroblasts (LIFs) (Figure 1C). The latter two populations have been studied mostly during the alveolar stage of lung development. Therefore, epithelial-mesenchymal interactions dictate and determine the morphogenic program during lung development, and most likely neomorphogenic programs during lung regeneration in adult life. In the next sections, we will discuss the involvement of mesenchymal cells in lung development as well as in normal (regeneration) versus aberrant repair (fibrosis) after injury in the adult, with a special emphasis on the function of the mesenchyme as a signaling hub for other cells, in particular, epithelial cells.

## Mesenchymal cell hierarchy and heterogeneity in lung development

In contrast to the lung epithelium, the lineage hierarchy of lung mesenchymal cells is poorly understood. Clonal analysis of mesenchymal progenitors during embryonic lung development has shown that a single mesenchymal progenitor cell expressing the early lung mesenchymal marker T-box transcription factor 4 (*Tbx4*) undergoes clonal expansion, and its daughter cells migrate to occupy distinct stromal niches where they influence epithelial cell behavior and morphogenic programs (28). Another report revealed that pulmonary mesenchyme also derives from multipotent cardiopulmonary progenitors that originate from the heart and give rise to pulmonary ASMCs/VSMCs, proximal vascular endothelium, and pericyte-like cells (29). Fibroblast growth factor 10 (*Fgf10*), a downstream target of TBX4 in the embryonic lung, is characterized by a distinctive expression pattern in the distal mesenchyme, facing epithelial bud tips that express the epithelial receptor fibroblast growth factor receptor 2-IIIb (*Fgfr2b*) and undergoing branching (27, 30) (Figure 1B). Using grafting experiments, it was initially shown that this distal mesenchymal tissue contains the necessary information and signals to induce epithelial budding and subsequent branching morphogenesis (31). Such instructive signals are believed to be largely mediated by FGF10/FGFR2b signaling (Figure 1B). In fact, genetic deletion of *Fgf10* or *Fgfr2b* leads to multiorgan agenesis, including the lung, as well as other developmental abnormalities (32–35).

FGF10 has long been believed to act as a chemoattractant, marking the future domain of epithelial tip outgrowth; therefore, its localized expression facing the growing epithelial buds has been assumed to be important for iterative branching and its associated stereotypy. However, this model has been challenged, as ubiquitous overexpression of *Fgf10* in the background of *Fgf10*-knockout pups was shown to rescue lung agenesis, yielding a seemingly normal branching pattern in the embryonic lung (36). Another characteristic of distal FGF10^+^ cells is that they migrate proximally to fit the growing epithelial tubes like a sleeve and give rise to the surrounding ASMC layer (37). This observation was later confirmed using genetic lineage tracing, and it was shown that mesenchymal FGF10^+^ cells are progenitors not only for ASMCs but also for VSMCs during the early pseudoglandular stage of lung development, and additionally for LIFs during embryonic stages and postnatally (34, 38–40). It is worth mentioning that ASMC peristalsis might also promote branching morphogenesis by constricting bud tips and creating clefts at bifurcation sites, although this concept is still debatable (26, 41, 42). Moreover, lineage tracing of mesenchymal cells expressing transcription factor 21 (*Tcf21*), another gene that marks LIFs during development and adulthood, showed a similar progenitor profile to FGF10^+^ cells, with TCF21^+^ cells featuring an SMC differentiation program during early development and a LIF program during later stages (43).

During postnatal lung development, the two most prominent mesenchymal populations are AMFs and LIFs (44) (Figure 1C). AMFs are α-smooth muscle actin–positive (ACTA2^+^) mesenchymal cells located at the alveolar entry ring and are believed to drive secondary septation, a process by which primitive alveolar sacs are subdivided into alveoli. The concept that secondary septation occurs via protrusion of alveolar walls toward the airspace to form finger-like crests has been challenged by a study showing that such secondary septa are an artifact of 2D imaging of thin lung sections. Previous studies had shown that AMFs are detected at the tips of secondary septa during the alveolar stage of lung development. More recently, it was shown using 3D imaging of thick lung sections that such secondary septa are rather ridges that subdivide alveolar sacs into smaller alveoli (45–48). For a more comprehensive overview of alveologenesis, we refer readers to other recently published reviews (49–52).

AMFs seem to transiently appear in the developing lung, and previous studies have suggested that they undergo apoptotic clearance upon completion of alveologenesis (53, 54). Recent work has demonstrated strong interaction between AMFs and AT1s particularly via wingless-related integration site (WNT) and sonic hedgehog (*Shh*) signaling (48). AMFs also signal to AT2 progenitors and influence their proliferation (55). Platelet-derived growth factor receptor-α (PDGFRα) has been identified as a marker for AMFs (45, 56–62), and the progenitors for AMFs have been shown to be positive for glioma-associated oncogene 1 (*Gli1*) (63, 64). GLI1 is a downstream effector and readout for *Shh* signaling, although it has been shown that it can be activated in a noncanonical fashion, such as by MAPK (65). Interestingly, although AMFs emerge in the lung during the alveolar stage of lung development between postnatal day 5 (P5) and P30 (Figure 1C), their GLI1^+^ progenitors are specified very early on during embryonic lung development (63).

**Figure 2:**
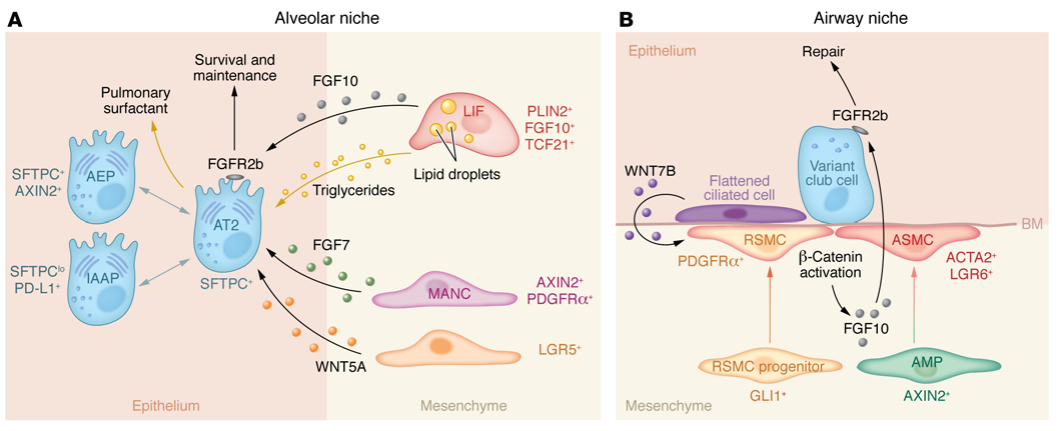
Mesenchymal-epithelial interactions in the alveolar and airway niches. (**A**) AT2 and their so-far identified subclusters are shown. Among the identified niche cells are LIFs, MANCs, and LGR5^+^ cells. Cellular markers are also shown. (**B**) A WNT-FGF feedback loop mediates epithelial-mesenchymal communication during airway regeneration. AEP, alveolar epithelial progenitor; AMP, AXIN2^+^ myofibrogenic progenitor; ASMC, airway smooth muscle cell; AT2, alveolar epithelial type 2; BM, basement membrane; Epi, epithelium; IAAP, injury-activated alveolar progenitor; LGR5, leucine-rich repeat containing G protein–coupled receptor 5; LIF, lipofibroblast; MANC, mesenchymal alveolar niche cell; Mes, mesenchyme; RSMC, repair-supportive mesenchymal cell.

LIFs, on the other hand, are lipid droplet–containing mesenchymal cells that are closely associated with AT2s (44, 66, 67) (Figure 1C and Figure 2A). They store triglycerides and transfer them to adjacent AT2s to be used during the production of pulmonary surfactant. LIFs are also a source of important growth factors, such as FGF10. FGFR2b signaling has been demonstrated to be important for the maintenance of AT2 identity and progenitor state (68–71) (Figure 2A). The AT2-supportive potential of LIFs has also been demonstrated ex vivo using alveolar organoids (72, 73). In contrast to AMFs, LIFs persist in the lung following alveolar maturation, and their role in repair after injury will be discussed in subsequent sections. Although the presence of LIFs in the human lung has been questioned (74–78), recent studies, including those using single-cell transcriptomics, have confirmed it (79–82).

LIFs were initially identified in rodents by the expression of adipose differentiation–related protein (*Adrp*), also called perilipin 2 (*Plin2*), a protein involved in lipid droplet trafficking (83, 84). As mentioned above, lineage-tracing studies established *Fgf10* and *Tcf21* as LIF markers in the mouse lung (38, 40, 43). With the advent of single-cell transcriptomics, other markers have emerged as more specific than *Plin2*, including, apart from *Fgf10* and *Tcf21*, LIM and calponin homolog domains 1 (*Limch1*), glycogenin (*Gyg*), microtubule-actin cross-linking factor 1 (*Macf1*), microfibril-associated protein 4 (*Mfap4*), nephronectin (*Npnt*), *Wnt2*, collagen type XIII α1 chain (*Col13a1*), and indolethylamine *N*-methyltransferase (*Inmt*) in mice and *LIMCH1*, α_2_-macroglobulin (*A2M*), regulator of cell cycle (*RGCC*), apolipoprotein E (*APOE*), and follistatin (*FST*) in humans (80, 81, 85, 86).

It is important to mention that the knowledge summarized above regarding branching morphogenesis, mesenchymal heterogeneity, and epithelial-mesenchymal crosstalk is predominantly based on research conducted in experimental rodent models, including transgenic mice. Corresponding data on human lung development are progressively emerging (87–94). For example, a comparative study highlighted differences in terms of expression patterns and biological activities of FGF ligands between mouse and human fetal lungs (90). Another study used distal tissues from prenatal human lungs to show that mesenchymal cells adjacent to bud tips express the WNT agonist R-SPONDIN 2 (RSPO2), which acts on its receptor leucine-rich repeat–containing G protein–coupled receptor 5 (LGR5) to maintain distal epithelial progenitors and their multipotency (91). Another study analyzed human terminal and respiratory bronchioles (TRBs) and fetal tissues to identity a novel population of bipotent alveolar type 0 (AT0) cells representing a transitional state as AT2s differentiate into AT1s or TRB secretory cells (94). This work also highlighted a population of LGR5^+^ fibroblasts enriched for WNT, PDGF, brain-derived neurotrophic factor (BDNF), and TGF-β signaling pathways, and serving as a source of FGF, bone morphogenetic protein (BMP), and WNT ligands that potentially signal to basal and secretory cells in the TRBs. The authors also identified the AT0 population in monkeys exposed to bleomycin (94). Human fetal lung atlases have also been recently published for 5 to 14 (87) and 5 to 22 weeks after conception (92).

## Mesenchymal-epithelial interactions in the injured lung

In recent years, FGF signaling has emerged as an important mediator of stem cell activation and subsequent epithelial repair in the lung (reviewed in ref. 95). FGF ligands such as FGF7 and FGF10 are typically secreted by mesenchymal cells and act on epithelial stem and progenitor cells to initiate the repair/regenerative process. This mechanism has been demonstrated in the context of both alveolar (Figure 2A) and airway epithelial regeneration (Figure 2B). WNT ligands, particularly WNT5A and WNT7B, were also shown to be instrumental in driving regenerative mechanisms in the lung (Figure 2). In the next subsections, the mesenchymal niche and its secreted factors will be discussed in the context of airway and alveolar repair and regeneration.

## The mesenchymal niche during airway epithelial regeneration

Club cells are dome-shaped, nonciliated secretory cells that are characterized by the expression of secretoglobin family 1A member 1 (SCGB1A1; also called Clara cell 10 kDa secretory protein CC10 or CCSP) and located in the conducting airways. These cells can give rise to both ciliated and secretory cells (96, 97). Because of their high expression of cytochrome P450 family 2 subfamily F polypeptide 2 (CYP2F2), club cells are selectively targeted and dramatically depleted by naphthalene (98–100). Interestingly, surviving variant club cells (CYP2F2^lo^) located at neuroepithelial bodies or bronchioalveolar duct junctions mediate the repair process in the bronchial epithelium (101–103) (Figure 2B). Accordingly, the naphthalene injury model is widely used to study mechanisms of club cell replenishment and airway regeneration in experimental mice.

Previous work has shown that following naphthalene injury, the majority of club cells are depleted, and ciliated cells flatten to cover the denuded epithelium and maintain barrier integrity (104, 105) (Figure 2B). These cells secrete the WNT ligand WNT7B, which acts on neighboring ASMCs to activate β-catenin signaling and FGF10 production. The latter acts on variant club cells expressing *Fgfr2b* to induce regeneration (105) (Figure 2B). It was also shown that *Lgr6* expression identifies a subset of ASMCs that promotes epithelial repair after naphthalene injury in a similar WNT-FGF10–mediated mechanism (106) (Figure 2B). Another mesenchymal population that is relevant in this context is AXIN2^+^ myofibrogenic progenitors, which have been shown to contribute to the ASMC lineage during repair after naphthalene injury (107) (Figure 2B). It is also important to mention that basal cells in cartilaginous airways are also involved in airway regeneration through a WNT7B-FGF10 axis that is regulated by the Hippo pathway (108).

Recently, a novel population of repair-supportive mesenchymal cells (RSMCs) was identified (109). These cells are distinct from ASMCs and mostly appear after naphthalene injury (Figure 2B). RSMCs derive from ACTA2-negative progenitors that transit through the SMC lineage and gain *Pdgfra* expression. When compared with the SMC-enriched fraction, the RSMC-enriched fraction displays superior ability to support club cell growth in the context of the bronchiolosphere assay (109, 110). The cellular origin of RSMCs that appear around the injured airway epithelium was further investigated (111). Lineage tracing of preexisting versus de novo–formed ACTA2^+^ cells as well as GLI1^+^ cells in the context of naphthalene injury showed that preexisting GLI1^+^ cells are a source of RSMCs in the lung (111) (Figure 2B). Along this line of evidence, genetic deletion of *Fgf10* in preexisting GLI1^+^ cells attenuates RSMC appearance and impairs club cell replenishment (111, 112). Bronchiolosphere assays confirmed the intrinsic ability of GLI1^+^ cells to support club cell growth (111). Further single-cell RNA sequencing (RNA-Seq) uncovered the cellular heterogeneity of GLI1^+^ cells in the healthy lung and suggested that alveolar fibroblasts might be an unexpected contributor to the airway mesenchymal niche (111). This finding is intriguing and goes against the dogma that mesenchymal niche cells are restricted to predefined anatomical locations in the adult lung; however, further studies are needed to determine whether these cells are capable of overcoming anatomical boundaries or whether they undergo reprogramming to resemble alveolar fibroblast–like cells that support epithelial regeneration.

## The mesenchymal niche during alveolar repair and regeneration

Although AT2s have traditionally been regarded as a homogenous pool of progenitor/stem cells in the adult lung, emerging literature suggests that they also contain diverse subsets. Initial ablation experiments have shown that AT2s that escape ablation undergo clonal expansion and replenish the AT2 pool, thereby restoring homeostasis (72). These studies also established that PDGFRα^+^ stromal cells, a population that includes LIFs in the adult lung, represent an important mesenchymal niche for AT2s (72). Subsequently, it was shown that WNT-responsive AT2s are unique in their ability to repair the alveolar compartment (20, 23) (Figure 2A). Treatment of both mouse and human WNT-responsive alveolar epithelial progenitors with FGF7 or FGF10 significantly enhanced their growth in vitro (20). WNT ligands, such as WNT5A (23), also identify the mesenchymal niche for WNT-responsive AT2 stem cells (Figure 2A). Interestingly, the AT2 niche activity has recently been shown to be enriched within the FGF10^+^ LIF fraction of resident mesenchymal cells (73).

Recent work has also identified a subset of AT2s displaying low levels of the AT2 transcriptomic signature compared with classical AT2s (7). These SFTPC^lo^ cells, termed injury-activated alveolar progenitors (IAAPs), were preferentially amplified in response to pneumonectomy, upregulated the AT2 signature, and were positive for programmed cell death ligand 1 (PD-L1 or CD274) (7). These cells replenished the mature AT2 pool upon genetic deletion of *Fgfr2b* in AT2s (70). IAAPs were also activated in response to bleomycin-induced pulmonary fibrosis, and therapeutic intervention with recombinant FGF10 further boosted their response and improved repair (113).

Apart from LIFs, mesenchymal alveolar niche cells (MANCs) that are WNT-responsive (AXIN2^+^) mesenchymal cells residing in the alveolar regions have also been reported (107). These cells were PDGFRα^+^ and supported AT2s by producing IL-6 and FGF7 (107) (Figure 2A). MANCs also expressed tropomyosin receptor kinase B (TRKB) and therefore responded to BDNF secreted by AT2s undergoing differentiation into AT1s after acute lung injury (114). MANCs responded to AT2-derived BDNF by secreting FGF7 to promote regeneration (114). Another identified alveolar niche population is LGR5^+^ mesenchymal cells, which support alveolar epithelial differentiation by producing WNT ligands such as WNT5A (106) (Figure 2A). While LIFs, MANCs, and LGR5^+^ fibroblasts contribute to the mesenchymal niche for AT2s, the extent of overlap among these populations is still not clear. It also remains to be seen whether these niche cells equally support various AT2 subsets such as AXIN2^+^ AT2s and/or IAAPs, or whether there are specialized niche cell subsets dedicated to maintenance and expansion of each AT2 subset. It is worth mentioning that mesenchymal cells are not only involved in epithelial repair and regeneration after major injury but are also involved in compensatory growth following pneumonectomy. In this context, it was shown that mesenchymal contraction is critical for re-septation during compensatory regrowth in post-pneumonectomy (47).

In addition to AT2-mediated alveolar regeneration, airway epithelial cells can also be deployed when AT2s are exhausted as a result of extreme injury such as in the case of highly pathogenic influenza virus infection. Such airway cells include a rare population of intrapulmonary p63^+^ progenitor cells that mostly lead to dysplastic repair featuring bronchiolarization of the alveolar regions and persistence of KRT5^+^ basal cell–like clusters (pods) rather than give rise to AT1s and AT2s and effective regeneration (reviewed in ref. 115). In this context, genetic deletion of *Fgfr2b* in SOX2^+^ cells before bleomycin injury (*Sox2* marks airway epithelial cells and not only intrapulmonary p63^+^ progenitors) inhibited the formation of KRT5^+^ pods and airway-derived AT2s (71). On the other hand, overexpression of *Fgf10* in these cells favored the AT2 fate over the KRT5^+^ pod fate, thus promoting fibrosis resolution and alveolar regeneration (71). β-Catenin stabilization in preexisting SOX2^+^ cells decreased the number of traced KRT5^+^ cells while enhancing the number of traced AT2s, again establishing the notion that WNT signaling favors AT2 differentiation (18). However, precise analysis of the mesenchymal niche that sways such fate decisions is still lacking. In a similar context, influenza virus inhibited β-catenin–mediated *Fgfr2b* expression in epithelial stem/progenitor cells (EpiSPCs), and exogenously applied recombinant FGF10 activated noninfected EpiSPCs and improves outcomes in infected mice (11). It is therefore clear that mesenchyme-derived signals, such as FGF10, are important components/effectors of the niche that influences scar-free regeneration versus dysplastic remodeling after major alveolar injury.

## Mesenchymal activation in the injured lung: complex outcomes

Orchestrated spatio-temporal activation of the mesenchyme is critical for adaptive repair and efficient regeneration of the lung, while a dysregulated mesenchymal response may lead to unremitting and uncontrolled repair responses that culminate in fibrosis. In most cases, it appears that the same developmental pathways that participate in normal repair are co-opted to promote pathological tissue responses. Identification of specific pathological mesenchymal cell populations that can be either reprogrammed or eliminated in concert with key signaling pathways and/or metabolic perturbations that drive fibrosis will be critical to developing novel and more effective therapies.

### Shh/GLI1 axis in mesenchymal activation and airway regeneration.

*Shh* signaling is one of the most studied developmental pathways. During embryonic lung development, *Shh* is expressed at high levels by the distal epithelium, and it signals through its mesenchymal receptor patched 1 (*Ptch1*) to induce mesenchymal proliferation (116), although autocrine *Shh* signaling in the developing trachea has also been reported (117). *Shh* signaling is also important for mesenchymal differentiation such as toward SMCs during lung development (118). In strong contrast to the embryonic scenario, *Shh* was shown to maintain mesenchymal quiescence in the adult mouse lung (119). Loss of epithelial *Shh* due to naphthalene injury led to decreased mesenchymal *Shh* activation but increased GLI1^+^ mesenchymal cell expansion (119). During injury resolution and epithelial regeneration, there was enhanced *Shh* activation and decreased mesenchymal proliferation (119). Forced activation of *Shh* in the mesenchyme impaired epithelial regeneration (119). GLI1^+^ cells also serve as a source of RSMCs that produce FGF10 needed for club cell replenishment and epithelial regeneration in response to naphthalene injury (111). Therefore, activation of GLI1^+^ cells is an integral part of the airway repair machinery, and paracrine signaling between this population and airway epithelial progenitors largely mediates the regeneration process. Interestingly, long-term fate mapping also showed that RSMC descendants were not completely cleared from the lung following the completion of airway regeneration (109). Moreover, such descendants did not contribute to myofibroblast formation if the animals were re-exposed to bleomycin as a second hit (109). Further research is needed to elucidate the long-term function of these cells and whether they might contribute to other pathological events such as airway remodeling.

### Aberrant mesenchymal activation in lung remodeling.

On the other hand, the literature clearly shows that aberrant mesenchymal activation, particularly that of the GLI1^+^ lineage, disrupts lung structure and can lead to fibrosis. GLI1^+^ cells, also regarded as perivascular mesenchymal stem cell–like (MSC-like) cells (120, 121), have been shown to be important contributors to fibrosis-associated myofibroblasts in multiple organs, including the lung (120, 121). Genetic ablation of these cells attenuated fibrosis in the kidney, heart, and bone marrow (120, 122). GLI1^+^ cells formed a pathological niche that skewed the differentiation of airway progenitors toward basal cell metaplasia instead of AT2 differentiation by antagonizing BMP signaling in the fibrotic lung (123). Ectopic hedgehog activation in distal fibroblasts led to loss of alveoli and airspace enlargement (124).

LIFs are another important contributor to fibrosis-associated myofibroblasts. Residing in close vicinity to AT2s, LIFs are naturally among the first responders to injury signals, which are largely profibrotic, released by AT2s and/or other alveolar cells. Earlier work had already shown that LIFs or LIF-like cells transdifferentiate into myofibroblasts in response to nicotine (125) or hyperoxia exposure (126). In studies using bleomycin to induce lung fibrosis in adult mice, LIFs were shown to give rise to fibrosis-associated myofibroblasts during fibrosis development (127). Interestingly, a recent report showed that, in addition to myofibroblasts, LIFs also display an augmented invasive, proliferative, contractile, and ECM-producing profile (128). Fibroblasts isolated from patients with idiopathic pulmonary fibrosis (IPF) exhibited an invasive phenotype that was dependent on hyaluronan synthase 2 (HAS2) and the hyaluronan receptor CD44 (129). This invasive IPF fibroblast phenotype is reminiscent of metastatic lung adenocarcinoma cancer cells (130). Importantly, the reverse differentiation trajectory (myofibroblast-to-LIF differentiation) occurs during fibrosis resolution (127). Fibrosis development and resolution are largely mediated by TGF-β1 and PPARγ signaling, respectively. In agreement with these findings, forced PPARγ activation in primary human lung fibroblasts attenuated TGF-β1–mediated fibrogenesis and promoted LIF formation (127). Therefore, myofibroblast-to-LIF transdifferentiation may represent an important route for myofibroblast deactivation and fibrosis resolution and could potentially be considered in future therapies to treat patients with progressive fibrotic disorders. Another recent study also described myofibroblast deactivation during the resolution phase (131). The authors identified aldehyde dehydrogenase 2 (*Aldh2*) and nuclear receptor subfamily 3 group C member 1 (*Nr3c1*) as potential antifibrotic genes that were downregulated at the peak of fibrosis and upregulated during fibrosis resolution (131). Apoptotic clearance is also a mechanism for fibrosis resolution in the lung, and it features the expression of proapoptotic markers such as the death receptor Fas (132, 133). The balance between myofibroblast deactivation and apoptosis during fibrosis resolution remains to be determined. It is also possible that both processes are critical to fibrosis resolution, as illustrated by suppression of the transcription factor Myo-D, which mediated myofibroblast dedifferentiation while also lowering the apoptosis threshold (134).

IPF is associated with metabolic disorders, and type 2 diabetes mellitus is a risk factor for developing this disease (134–137). Fibrosis-associated myofibroblasts displayed an altered bioenergetic profile where inactive adenosine monophosphate–activated protein kinase (AMPK) in these cells promoted their persistent activation by decreased autophagy, increased ECM production, mitochondrial dysfunction, and resistance to apoptosis (138). Restoring AMPK activity in such myofibroblasts improved mitochondrial biogenesis and enhanced autophagy, ECM turnover, and sensitivity to apoptosis, thus leading to myofibroblast deactivation (138). Notably, the first-line antidiabetic compound metformin, a known AMPK agonist, has proved to be effective in reversing lung fibrosis in the mouse bleomycin model via this mechanism (138). Interestingly, such a beneficial effect of metformin was independently validated when its administration accelerated fibrosis resolution by promoting myofibroblast-to-LIF transdifferentiation (139). The latter study also shed light on an additional AMPK-independent mechanism that leads to BMP2 release and PPARγ activation (139).

Heterogeneity of lung-resident mesenchymal cells in response to influenza virus infection has recently been investigated (140). The authors identified a subset of damage-responsive fibroblasts, expressing the ECM protease ADAMTS4, that aggravates the immune response and leads to structural and functional impairment of the lung (140). Although the immunomodulatory roles of mesenchymal cells, particularly those of MSCs, have already been reported (reviewed in ref. 141), niche–progenitor cell interactions in the context of influenza-induced acute respiratory distress syndrome or even SARS-CoV-2 are still largely unexplored.

### Effects of mesenchymal aging on lung fibrosis.

Lung aging that may involve both cellular and noncellular components of the stem cell niche, particularly the ECM, adversely affects lung regenerative capacity, thus predisposing to chronic lung diseases such as IPF and chronic obstructive pulmonary disease. Previous studies have identified aging as a critical determinant of the lung’s ability to resolve fibrotic injury (142–145). While senescent cells have been shown to accumulate in aging tissues (146, 147), their role in age-related diseases has been debated. Considerable heterogeneity exists between senescent cells across tissues that may be related to their physiologically programmed, preexisting transcriptomic signatures and their unique cellular microenvironments; another important contribution to this heterogeneity lies in differences of the senescence-provoking stimuli. For example, oxidative stress–induced senescence in young mice is often transient and may even support physiological repair through a pro-regenerative senescence-associated secretory phenotype (SASP) profile (148), while senescence induced by the same stimulus in aged mice may confer a persistent/progressive, pathological response (142). Such differences highlight the importance of defining the heterogeneity and functional characteristics of cells that acquire a growth-arrested state with expression of the widely used senescence marker *p16^INK4a^*. Differences in the context and timing of elimination of *p16^INK4a^*-expressing cells may also explain differences in their (patho)physiological roles. For example, there are important differences in the outcome of the injury-repair process dependent on whether the intervention prevents the formation of senescent cells versus the elimination of senescent cells that accumulate during pathological disease states (149–151).

Studying the role of senescence and aging in lung diseases has further illuminated the importance of mesenchymal-epithelial crosstalk within the stem cell niche. The elimination of *p16^INK4a^*-expressing cells after established bronchopulmonary dysplasia in a murine hyperoxia model led to improved lung regeneration in association with increased numbers of LIFs and AT2s (151). In an ex vivo alveolosphere-organoid model, aging of the mesenchymal component was critical to AT2 proliferation and alveolosphere formation (152). This inability of mesenchymal cells to support AT2 cell proliferation and differentiation is linked to acquisition of senescence features and metabolic reprogramming, in part, related to elevated expression of the reactive oxygen species–generating enzyme NADPH oxidase 4 (NOX4). Epigenetic targeting of NOX4 with an inhibitor of bromodomain-containing protein 4 (BRD4) accelerated fibrosis resolution in an aging murine model of lung injury (153). In contrast to the pro-senescent, pro-oxidant, and profibrotic actions of NOX4 (154, 155), the augmentation of the antioxidant, antiinflammatory, and senolytic effects of the mitochondrial protein deacylase sirtuin-3 (SIRT3) on macrophages and fibroblasts was effective in restoring pro-regenerative effects in aged mice (145). Mitochondrial dysfunction has been implicated in both AT2 and mesenchymal cell senescence and aging (156, 157). Recent studies have implicated a role for uncoupling protein 2 (UCP2) in loss of mitochondrial bioenergetics, deficient fatty acid oxidation, and senescence of fibroblasts that may account for a nonresolving, persistent/progressive phenotype in aging (158). Thus, like lung development and homeostasis during adulthood, the metabolic and epigenetic programming of the mesenchyme during aging has a critical role in determining the outcome of repair/regenerative responses to lung injury.

## Future directions and clinical implications

Recent and emerging studies highlight the importance of balanced mesenchymal activation and fate determination in the lung. Transient activation seems to initially occur in response to injury, and this event primes the lung to undergo regeneration and restore barrier integrity and respiratory function. On the other hand, dysregulated mesenchymal activation appears to be a driver of aberrant repair and fibrosis. The reversibility of injury and the robust reparative capacity observed in model systems have allowed the studying of “scarless” regenerative mechanisms in the mammalian lung.

One issue that urgently needs to be addressed by the scientific community is the integration of omics data published by various research groups and consortia, especially on single-cell RNA-Seq and spatial transcriptomics, into a comprehensive lung mesenchymal cell atlas. Such an atlas should not only list known and novel cell types during homeostasis, disease, and regeneration but also (a) deconvolute mesenchymal cell identity by demarcating stable versus transient cell states, (b) uncover the spatial, functional, and molecular overlap between published cell types and states, and (c) standardize/unify nomenclature. For example, the term “lipofibroblast” as we have used it in this Review is not consistently used in the literature; rather, these cells are often lumped into the designation of “alveolar fibroblasts” or “fibroblasts” as described in several single-cell RNA-Seq data sets (69, 88, 94, 148, 159–161). Although such efforts at standardization have already begun to gain interest within the community, a focused endeavor to develop consensus nomenclature is strongly warranted (also addressed in ref. 162). It is important to mention here that recent advancements in lineage-tracing tools such as the use of split-Cre, Cre/flippase, and Cre-ERT2/Dre-ERT2 dual recombinase approaches will certainly improve data interpretation in emerging research. These approaches, coupled with the increasing bioinformatics input into the field, will provide a platform to achieve better understanding of mesenchymal cell subsets and states under various settings.

Another issue is the transferring of knowledge from the mouse model system to the human context. Interestingly, recent work has highlighted some disparities between mouse and human lung cell biology and airway morphology. For example, it was shown that, unlike mouse AT2s, human AT2s give rise to basal cells during fibrotic remodeling (163). The use of novel tools such as induced pluripotent stem cell–derived cell lines and organoids, precision-cut lung slice cultures, and humanized injury models represents a step forward toward reconciling the concepts and paradigms established using mouse models in preclinical studies.

As discussed in this Review, the interdependence of the epithelium and mesenchyme in maintenance of homeostasis and repair of adult tissues has important clinical implications. The recognition that mesenchymal plasticity determines and drives epithelial repair and regenerative responses implies that health and resilience of tissues/organs will depend on assuring mesenchymal cell responses that are spatio-temporally regulated. Furthermore, in fibrotic diseases affecting diverse organs, therapeutic targeting of tissue-resident fibroblasts/mesenchymal cells to promote niche-supporting regenerative phenotypes, while reprogramming or eliminating fibrosis-perpetuating phenotypes, will lead to innovative new therapies for this recalcitrant, debilitating, and ultimately fatal group of disorders. The challenge remains to exploit advancing knowledge of mesenchymal plasticity and to innovate new therapeutic strategies that promote regeneration of the lung to prevent, retard, and even reverse fibrosis.

## Version 1 07/17/2023

Electronic publication
